# Electric-field-induced Spontaneous Magnetization and Phase Transitions in Zigzag Boron Nitride Nanotubes

**DOI:** 10.1038/srep12416

**Published:** 2015-07-24

**Authors:** Lang Bai, Gangxu Gu, Gang Xiang, Xi Zhang

**Affiliations:** 1 College of Physical Science and Technology, Sichuan University, Chengdu 610064, China; 2 College of Materials Engineering, Panzhihua University, Panzhihua 617000, China; 3 Key Laboratory of High Energy Density Physics and Technology of Ministry of Education, Sichuan University, Chengdu 610064, China

## Abstract

We demonstrate an alternative scheme for realizing spin polarizations in semiconductor nanostructures by an all-electric way. The electronic and magnetic properties of the model system, zigzag pristine boron nitride nanotubes (BNNTs), are investigated under a transverse electric field (*E*) through spin-polarized density functional theory calculations. As *E* increases, the band gap of BNNTs is reduced due to charge redistribution induced by the asymmetry of electrostatic potential energy, and BNNTs experience rich phase transitions, such as semiconductor-metal transition and nonmagnetic (NM) metal-ferromagnetic (FM) metal transitions. Electric-field-induced magnetization occurs when a sufficiently high density of states at the Fermi level in the vicinity of metal-insulator transition is reached due to the redistribution of electronic bands and charge transferring across the BNNTs. Further analysis show that the spontaneous magnetization is derived from the localized nature of the 2*p* states of B and N, and the ferromagnetic coupling is stabilized by Zener’s double-exchange mechanism. Our results may provide a viable way to realize spintronic devices for applications.

Since the spin field-effect transistor (SpinFET) was first proposed by Datta and Das in 1990^1^, many efforts have been put into the search for reliable mechanisms for manipulations of spin polarizations, including spin injection, spin transport and spin detection, in semiconductors. Up to now, three main schemes have been proposed: (1) fabrication of hybrid structures combining ferromagnetic (FM) metals with nonmagnetic (NM) semiconductors^2^,^3^; (2) synergy of magnetism and semiconducting properties in the same materials, i.e., FM semiconductors^4^,^5^,^6^; (3) generation of spin-polarized currents via spin-orbit coupling^7^,^8^. While the first two include magnetic materials which may generate stray fields or have other disadvantages for applications, such as conductivity mismatch^9^ or low Curie temperature problem^10^, the third one could be all-electric which is desirable for the applications of spintronic devices such as SpinFETs^11^,^12^. Recently, an all-electric SpinFET using asymmetrically biased quantum point contacts was first demonstrated by T. M. Chen *et al.*^13^, in which an effective magnetic field is generated by an local electric field from the pair of nano-scale gates via the spin-orbit coupling^14^.

In this letter, we will demonstrate based on first-principles calculations that an alternative all-electric approach can be used to induce spin polarization in the model system of semiconductor nanostructures, boron nitride nanotubes (BNNTs), in which the mechanism is rooted in that the reconstruction of electronic bands induced by an applied electric field, as well as the spin polarized nature of 2*p* electrons and holes after the reconstruction, gives rise to a spontaneous magnetism. As is well known, pristine BNNTs are NM materials, while the magnetic properties can be modified by structural modifications of BNNTs. Recent theoretical advances demonstrate that magnetization of BNNTs can be tuned by doping various atoms (O, C, Ge, Be, Cr, Mn, *et al.*^15^,^16^,^17^), carrier injection^18^, and adsorption of H atoms^19^ and F atoms^20^. Here, without those structural engineering, we will demonstrate that applying a transverse electric field *E* can induce spontaneous magnetization in pristine BNNTs, which may be desirable for the applications of spintronic devices. Meanwhile the rich phase transitions that the pristine BNNTs experiences, for instance, semiconductor-metal transitions and NM metal-FM metal transitions, will also be explored and discussed.

## Methods

The present calculations are performed using Vienna *ab initio* simulation package (VASP)^21^ which is based on density functional theory (DFT) with generalized gradient approximation (GGA) of Perdew-Burke-Ernzerhof (PBE)^22^ for the exchange correlation potential, as it is proved that local density approximation (LDA) is always poor for BN or GaN semiconductors^23^,^24^,^25^. The projector augmented wave (PAW)^26^ formalism is used to describe the core-valence electron interactions, and the kinetic energy cutoff for the plane-wave basis set is 500 eV. The gamma mesh 1 × 1 × 7 k-point is used for the Brillouin zone integration during the geometry relaxations and static calculations, which has been carefully checked. The structures of BNNTs are fully optimized (both lattice parameters and atomic positions) until the atomic forces are less than 0.01 eV/Å.

## Results and Discussion

**Figure 1:**
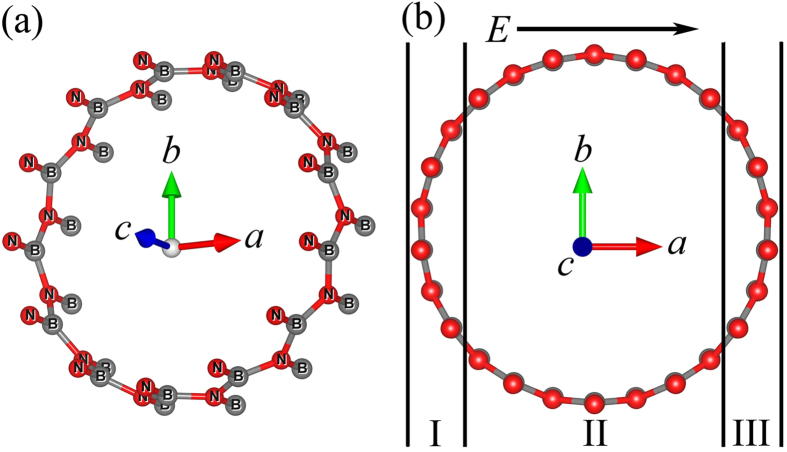
(**a**) Diagram of 130BNNT, red and dark grey spheres represent nitrogen atoms and boron atoms, respectively. (**b**) Cross-section view of 130BNNT. External transverse electric field *E* is in the direction of axis *a*.

The nanotube we studied, a (13, 0) chiral vector zigzag BNNT (130BNNT), is about 1 nm in diameter, and is geometrically constructed using a similar procedure described elsewhere^27^. The diagram of unit cell is shown in Fig. 1(a). In order to avoid interactions between nanotubes, we insert about 20 Å vacuum layers between adjacent BNNTs’ surfaces. Fig. 1(b) shows the scheme of 130BNNT that is looked along c direction. For convenience of discussion, 130BNNT has been divided into three regions: both region I and region III include 6 boron atoms and 6 nitrogen atoms, region II includes 14 boron atoms and 14 nitrogen atoms. The external transverse electric field (*E*) is applied along direction *a*.

**Figure 2:**
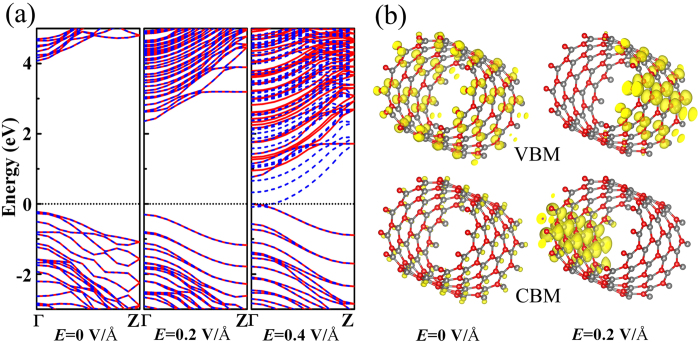
(**a**) Spin-resolved band structures of 130BNNT under different electric fields. Solid red lines represent spin-up channels, spin-down channels are denoted by dash blue curves. The Fermi-level is set to 0 eV. (**b**) Partial charge density of 130BNNT for VBM and CBM state under different electric field. The isovalue is 0.005 e/Å^3^.

We first calculated spin-polarized band structure of 130BNNT without E applied, as shown in Fig. 2(a). In agreement with previous calculations, 130BNNT is a NM, direct wide-gap semiconductor, the band gap is 4.30 eV, and both spin-up and spin-down channels are degenerated. The charge density of valence band maximum (VBM) and conduction band minimum (CBM) are plotted in Fig. 2(b). Charge density of VBM state is localized around all nitrogen atoms, and charge density of CBM state is distributed around boron atoms.

When the external field is applied and *E* = 0.2 V/Å, spin-up and spin-down channels are still degenerated, but the band gap of 130BNNT decreases to 2.66 eV. The reduction of band gap is attributed to charge redistribution, which result from the asymmetry of electronic potential. This phenomenon is also known as the giant stark effect^28^,^29^. Under E = 0.2 V/Å, charge density of CBM state move in the direction opposite to *E* to region I and localize around boron atoms, charge density of VBM state move along the electric field to region III and accumulate around nitrogen atoms (Fig. 2(b)). Because the electrostatic potential energy for electrons increases along the direction of *E*, the valence band edge (VBE) moves up and the conduction band edge (CBE) moves down, giving rise to the band gap reduction. When the external field continues increasing and *E* = 0.4 V/Å, the band gap is closed and 130BNNT becomes a FM metal with the total energy only lower than that of the NM state by 7 meV.

**Figure 3:**
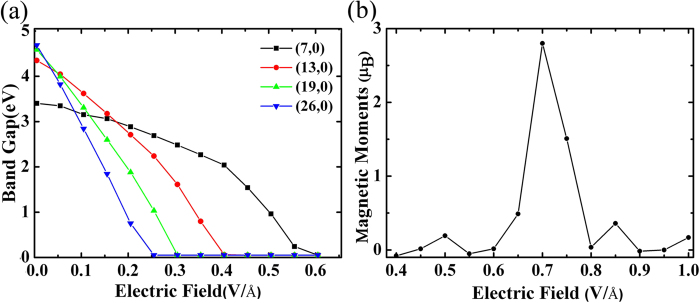
(**a**) Band gaps as a function of electric field strength for zigzag BNNTs with different chiral vectors. (**b**) Magnetic moments as function of electric field strength for 130BNNT.

The variation of the band gap values with the strength of external electric field applied on BNNTs is shown in Fig. 3(a). For comparison, we calculated the variation of band gaps of (7, 0), (19, 0) and (26, 0) zigzag BNNT whose diameter is 0.5 nm, 1.5 nm and 2 nm, respectively. Without electric field applied, BNNTs with smaller diameter have narrower band gaps because the hybridization effect is more important for smaller diameter BNNTs as the increase of tube curvature^30^. As shown in Fig. 3(a), the reduction of band gap for a given transverse electric field strength is larger for a larger diameter BNNTs. This is because VBM and CBM state are separated further from each other in the electrostatic potential field for larger diameter BNNTs which makes VBE shift up and CBE shift down more dramatically.

The most interesting thing is that spontaneous magnetic moments of BNNTs are induced and modulated by *E* after the band gap closure. The magnetic moment of 130BNNT under *E* is shown in Fig. 3(b). The appearance of magnetic moment is not apparent until *E* reaches 0.6 V/Å. As *E* increases from 0.6 V/Å, the magnetic moment increases rapidly, and 130BNNT becomes a FM metal. When the field reaches 0.7 V/Å, the ground state of 130BNNT is calculated to be FM with magnetic moments of 2.80 μ_B_ per unit cell, as the total FM energy is 158.2 meV lower than that of the NM state. After *E* > 0.7 V/Å the magnetic moment of 130BNNT decreases and vanishes. When *E* = 0.8 V/Å, 130BNNT becomes NM again.

**Figure 4:**
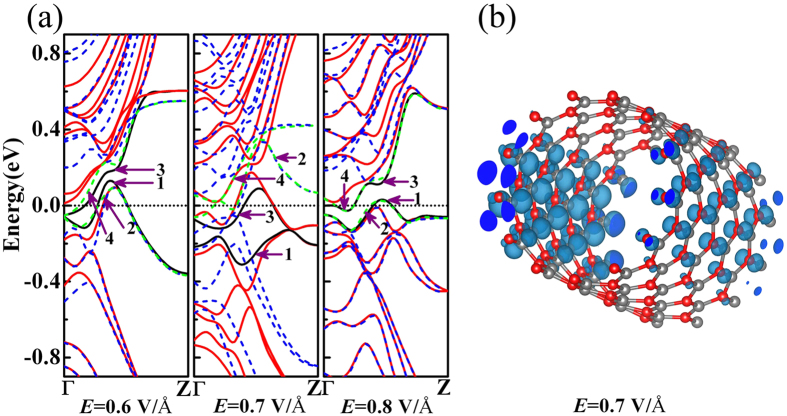
(**a**) Spin-resolved band structures of 130BNNT under different electric fields: Solid lines and dash curves represent spin-up and spin-down channels, respectively. The black solid lines which arrow 1 and arrow 3 point at are the spin-up channels of the top band of VB and bottom band of CB, respectively. The green dash curves which arrow 2 and arrow 4 point at are the spin-down channels of the top band of VB and bottom band of CB, respectively. (**b**) Spatial magnetization density of 130BNNT when *E* = 0.7 V/Å. The isovalue is 0.005 e/Å^3^.

To further investigate the magnetism in BNNTs with *E* applied, spin-resolved band structures and spatial magnetization density are shown in Fig. 4. When *E* reaches 0.6 V/Å, which is larger than the critical value for the band gap closure, both spin-up and spin-down channels of VBE and CBE are mixed together (Fig. 4(a)). As a result, electron transferring occurs and electrons on the states of VBE higher than the Fermi level transfer to the states of CBE lower than the Fermi level. However, spin-up and spin-down states of CBE below the Fermi level are still almost the same in quantity, so 130BNNT keeps NM. When *E* = 0.7 V/Å, parts of some spin-down VBE states move up higher than the Fermi level but all spin-up states of VBE are still lower than Fermi level, on the other hand, parts of some spin-up CBE states shift down to underside of the Fermi level, but all spin-down states of CBE still higher than the Fermi level. The electrons on the spin-down VBE states with energy higher than Fermi-level transfer to the spin-up states of CBE with energy lower than the Fermi level, so the amount of electrons with spin-up nature is greater than those with spin-down nature. This transferring induces the appearance of spontaneous magnetic moments, resulting in the FM of 130BNNT. The spatial magnetization density of 130BNNT under *E* = 0.7 V/Å in Fig. 4(b) shows that the magnetization density is mostly distributed around boron atoms in region I and nitrogen atoms in region III, while nitrogen atoms in region II have a little contribution. When *E* = 0.8 V/Å, since both spin-up and spin-down *2p* electrons on the VBE states higher than Fermi-level transfer to the CBE states lower than Fermi-level, the quantitative difference of spin-up and spin-down electrons below Fermi-level are almost zero, 130BNNT becomes NM again.

**Figure 5:**
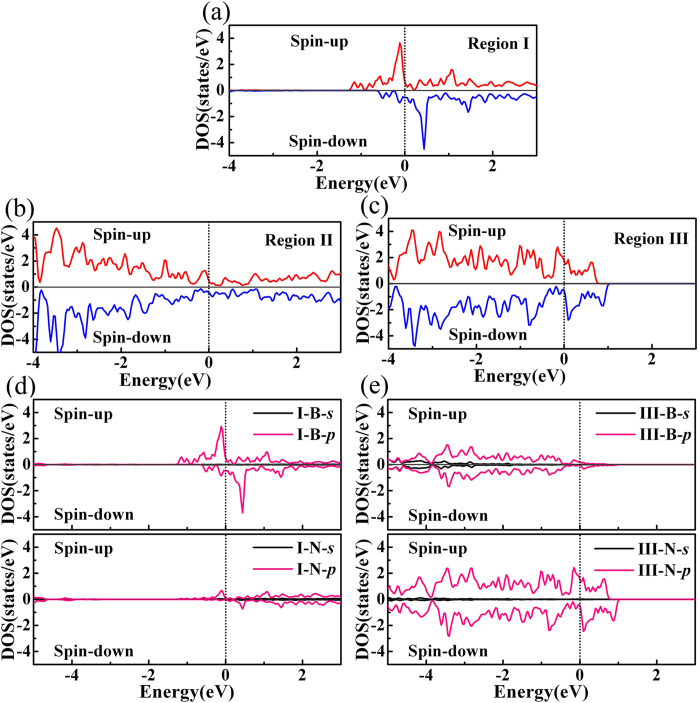
Spin-polarized DOS for (**a**) region I, (**b**) II and (**c**) III under *E* = 0.7 V/Å. (**d**) Comparison of *2s* and *2p* orbital partial DOS of N and B atoms for region I under *E* = 0.7 V/Å. (**e**) Comparison of *2s* and *2p* orbital partial DOS of N and B atoms for region III under *E* = 0.7 V/Å. The Fermi-level is set to 0 eV.

Since the magnetization is mostly attributed to the states near the Fermi level, we plot the near-Fermi-level partial density of states (PDOSs) of 130BNNT under *E* = 0.7 V/Å. As shown in Fig. 5(a–c), PDOSs of region I, region II and region III indicate that the majority of quantitative difference between spin-up and spin-down states near the Fermi level originate from region I and III, and the magnetization is mostly contributed by region I and III. The contributions of *2s* orbitals and *2p* orbitals of boron atoms and nitrogen atoms in region I and III on DOS are shown in Fig. 5(d,e). In region I, the major contribution of quantitative difference between spin-up and spin-down DOS is from *2p* orbitals of boron atoms, while little from *2p* orbitals of nitrogen atoms. On the other hand, in region III, *2p* orbitals of nitrogen atoms contribute mostly to spin polarization, while those of boron atoms contribute a little. The spin polarization of *2p* holes and electrons, which results from the previously discussed electron transferring under *E* after the band gap closure, is derived from the localized nature of the 2*p* states of nitrogen and boron and the magnetism in nature belongs to *d*^*0*^ magnetism^31^,^32^.

The occurrence of magnetism in BN nanotubes can be firstly evaluated by the Stoner criterion, which is described as the inequality *N*(*E*_*F*_) *I*_*S*_ > 1, where *N*(*E*_*F*_) is the DOS at the Fermi level and *I*_*S*_ is the Stoner parameter^33^,^34^. Since *I*_*S*_ equals to ∆/m, where ∆ is the exchange splitting of the bands and *m* is the magnetic moment in units of Bohr magneton (*μ*_*B*_)^35^, the Stoner parameter *I*_*S*_ can be estimated from our DFT calculations. For instance, for N atoms of 130BNNT in Region III at *E* = 0.7 V/Å, we find that ∆ = 0.242 eV, and *m* = 0.476 *μ*_*B*_/unit cell, so that the Stoner parameter *I*_*S*_ is estimated as 0.508 eV; meanwhile, *N*(*E*_*F*_) for N atoms is 2.09 states/eV/spin, as shown in Fig. 5(c). Therefore, the Stoner inequality is satisfied and the magnetism occurs. However, in order to reveal the type of the FM coupling, we need to look into spin-dependent electronic bands further. According to the work by M. Seike *et al.*^32^,^36^,^37^,^38^,^39^,^40^, localized bands, such as deep-impurity bands, must be partially occupied to stabilize *d*^*0*^ magnetism by Zener’s double-exchange mechanism. By analyzing the DOSs of BN nanotubes, we can see the similarity in localization between the impurity bands and the redistributed bands induced by the electric field. As Fig. 5(e) shows, under *E* = 0.7 V/Å, the 2*p* states of the N atoms near the Fermi level in region III are highly localized, giving rise to the localized magnetic moments on the N atoms. Since the localized 2*p* states near the Fermi level are partially occupied and the two N atoms with a distance of 2.5 Å are separated by one B atom, the FM coupling between the N atoms in region III is stabilized by Zener’s double-exchange mechanism^32^,^36^,^37^,^38^,^39^,^40^.

The Curie temperature (T*c*) in hole or electron-doped semiconductors such as BN usually is lower than the room temperature due to the short-range magnetic exchange interaction in the systems^32^,^38^,^39^. Interestingly, the electric-field-induced magnetism may provide a way for optimizing T*c*. Our work shows that carrier transferring is induced by the electric field, which eventually gives rise to the *d*^*0*^ ferromagnetism in BN nanotubes. Since the magnetism is directly induced by the electric field, the corresponding magnetic properties, such as magnetization and T*c*, can be tuned via changing electric field on BN nanotubes, which is different from the case of dopant-induced magnetism in which dopants tend to be fixed. Therefore, T*c* in BN nanotubes may be optimized by adjusting the electric field.

## Conclusion

In summary, we investigate the effect of a transverse electric field *E* on the electronic and magnetic properties of BNNT using first-principles calculations in this work. Not only can the band gap of BNNTs be modulated due to the asymmetry of charge redistribution under *E*, but also can the spontaneous magnetization be induced by *E*, which is resulted from the reconstruction of electronic bands and spin polarized nature of *2p* holes and electrons. Since the electric field *E* is easy to be applied and unapplied in practice, our results provide an all-electric way to modulate the spin-dependent properties of the semiconductor nanostructures and may be used for spintronic devices for applications.

## Additional Information

**How to cite this article**: Bai, L. *et al.* Electric-field-induced Spontaneous Magnetization and Phase Transitions in Zigzag Boron Nitride Nanotubes. *Sci. Rep.* **5**, 12416; doi: 10.1038/srep12416 (2015).
